# Diversity of *Hepatozoon* species in wild mammals and ticks in Europe

**DOI:** 10.1186/s13071-022-05626-8

**Published:** 2023-01-24

**Authors:** Mathilde Uiterwijk, Lea Vojta, Nikica Šprem, Ana Beck, Daria Jurković, Marja Kik, Georg G. Duscher, Adnan Hodžić, Slaven Reljić, Hein Sprong, Relja Beck

**Affiliations:** 1 Centre for Monitoring of Vectors (CMV), Netherlands Institute for Vectors, Invasive plants and Plant health (NIVIP), Netherlands Food and Consumer Product Safety Authority (NVWA), Wageningen, the Netherlands; 2 Division of Molecular Biology, Laboratory for Molecular Plant Biology and Biotechnology, Rudjer Boskovic Institute, Zagreb, Croatia; 3 Department of Fisheries, Apiculture, Wildlife Management and Special Zoology, Faculty of Agriculture, University of Zagreb, Zagreb, Croatia; 4 Department of Veterinary Pathology, Faculty of Veterinary Medicine, University of Zagreb, Zagreb, Croatia; 5 Laboratory for Parasitology, Department for Bacteriology and Parasitology, Croatian Veterinary Institute, Zagreb, Croatia; 6 Faculty of Veterinary Medicine, Dutch Wildlife Health Centre, Utrecht University, Utrecht, the Netherlands; 7 Austrian Agency for Health & Food Safety (AGES), Vienna, Austria; 8 Centre for Microbiology and Environmental System Science (CMESS), Department of Microbiology and Ecosystem Science, Division of Microbial Ecology (DoME), University of Vienna, Vienna, Austria; 9 Department of Forensic and State Veterinary Medicine, Faculty of Veterinary Medicine, University of Zagreb, Zagreb, Croatia; 10 Centre of Infectious Disease Control of the National Institute for Public Health and the Environment (Cib-RIVM), Bilthoven, the Netherlands

**Keywords:** Tick-borne diseases, Apicomplexa, Wildlife, Wild carnivores, Wild ungulates, Rodents, 18S ribosomal DNA

## Abstract

**Background:**

*Hepatozoon* spp. are tick-borne parasites causing subclinical to clinical disease in wild and domestic animals. Aim of this study was to determine *Hepatozoon* prevalence and species distribution among wild mammals and ticks in Europe.

**Methods:**

Samples of wild mammals and ticks, originating from Austria, Bosnia and Herzegovina, Croatia, Belgium and the Netherlands, were tested with PCR to amplify a ~ 670-bp fragment of the small subunit ribosomal RNA gene.

**Results:**

Of the 2801 mammal samples that were used for this study, 370 (13.2%) tested positive. *Hepatozoon* *canis* was detected in samples of 178 animals (3 Artiodactyla, 173 Carnivora, 1 Eulipotyphia, 1 Lagomorpha), *H.* *martis* in 125 (3 Artiodactyla, 122 Carnivora), *H.* *sciuri* in 13 (all Rodentia), *Hepatozoon* sp. in 47 (among which *Hepatozoon* sp. Vole isolate, all Rodentia) and *H.* *ayorgbor* in 4 (all Rodentia). Regarding origin, 2.9% (6/208) tested positive from Austria, 2.8% (1/36) from Bosnia and Herzegovina, 14.6% (173/1186) from Croatia and 13.9% (190/1371) from Belgium/the Netherlands. Of the 754 ticks collected, 0.0% (0/35) *Hyalomma* sp., 16.0% (4/25) *Dermacentor* spp., 0.0% (0/23) *Haemaphysalis* spp., 5.3% (24/50) *Ixodes* and 1.4% (3/221) *Rhipicephalus* spp. tested positive for *Hepatozoon* (4.2%; 32/754), most often *H.* *canis* (*n* = 22).

**Conclusions:**

*Hepatozoon* *canis* is most present in mammals (especially in Carnivora such as gray wolves and golden jackals) and ticks, followed by *H.* *martis*, which was found merely in stone martens and pine martens. None of the rodent-associated *Hepatozoon* spp. were detected in the ticks, suggesting the possible implication of other arthropod species or non-vectorial routes in the transmission cycle of the hemoprotozoans in rodents. Our findings of *H.* *canis* in ticks other than *R.* *sanguineus* add to the observation that other ticks are also involved in the life cycle of *Hepatozoon*. Now that presence of *Hepatozoon* has been demonstrated in red foxes, gray wolves, mustelids and rodents from the Netherlands and/or Belgium, veterinary clinicians should be aware of the possibility of spill-over to domestic animals, such as dogs.

**Graphical Abstract:**

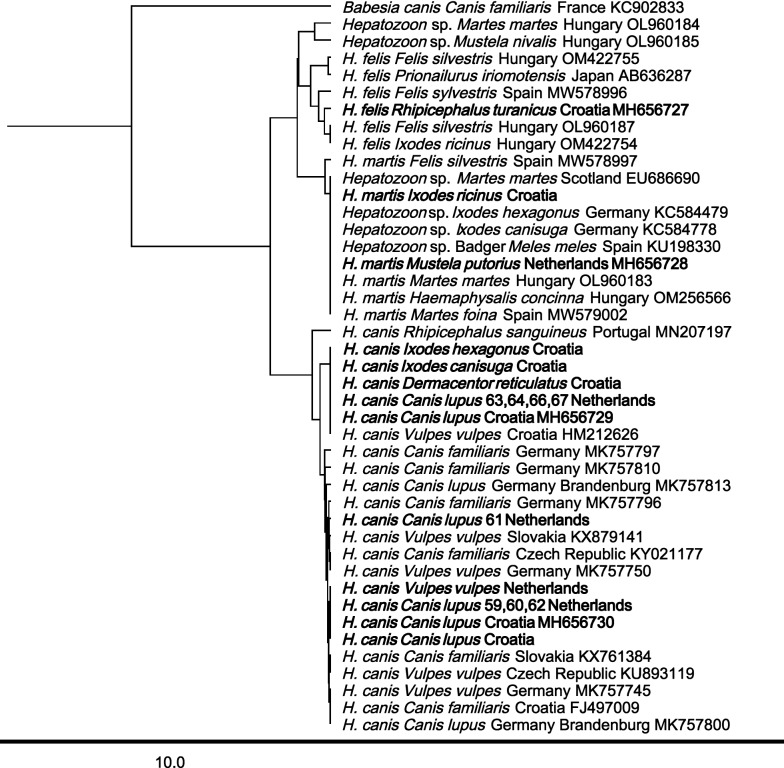

**Supplementary Information:**

The online version contains supplementary material available at 10.1186/s13071-022-05626-8.

## Background

*Hepatozoon* spp. (Adeleorina: Hepatozoidae) are apicomplexan vector-borne blood parasites with a complex life cycle [1, 2]. Vertebrates serve as intermediate hosts, and hematophagous arthropods such as ticks, mites and fleas serve as both definitive hosts and vectors [3–6]. Unlike other vector-borne pathogens, *Hepatozoon* transmission is not achieved by arthropod blood feeding on the vertebrate host, but with the infection taking place when the intermediate host ingests the definitive host. Also, transmission routes other than vector-borne have been described. For some *Hepatozoon* species, such as *H.* *americanum* (canids), *H.* *sipedon* (reptiles), *H.* *caimani* (caiman crocodiles) and *H.* *ayorgbor* (snakes), transmission can take place via predation of prey [7–12] or, in case of *H.* *canis* (canids) and *Hepatozoon* sp. of garter snakes (*Thamnophis* *elegans*), vertical transmission has been described [13–16].

The pathogenicity of *Hepatozoon* in wild animals such as canids [17, 18], felids [19] and mustelids [20] seems to be low, although in the case of co-infections with for example bacteria, severe disease manifestations may occur [21]. The importance of *Hepatozoon* in wild animals is mainly spillover to domestic animals. *Hepatozoon* infection in dogs and cats is known to cause subclinical to severe disease, which can be worsened by co-infection with, e.g., bacteria or other hemoparasites as well [2, 22–31].

Until a decade ago, histological methods prevailed in the characterization of blood parasites, with few exceptions [32–36]. Since around the 2010s, molecular methods have been commonly used [37], more specifically amplification of the small subunit ribosomal RNA (18S rRNA) gene fragments in case of detection and species identification of *Hepatozoon* infections. Several *Hepatozoon* species have been described in European wild and domestic mammals with these methods, with most studies focusing on carnivores [38, 39], e.g. *H.* *silvestris* and/or *H.* *felis* in European wild cats (*Felis* *silvestris* *silvestris*) in Bosnia and Herzegovina [19, 40], Spain [41] and Hungary [42], and in domestic cats from Italy [23, 43]. Also, *H.* *martis* has been detected in mustelids from Bosnia and Herzegovina and Croatia [44], Hungary [42] and Spain [41] and in wild cats from Spain [41, 45], *H.* *ursi* in brown bears (*Ursus* *arctos*) from Turkey [46] and *Hepatozoon* sp*.* in pine martens from Scotland, UK [20]. In wild canids such as red foxes (*Vulpes* *vulpes*) [17, 18, 41, 47, 48], gray wolves (*Canis* *lupus*) [49, 50] and golden jackals (*Canis* *aureus*) [51], prevalence of *H.* *canis* can be high. In contrast, prevalence of *H.* *canis* in domestic dogs [52, 53] and cats [39, 43] is generally (much) lower. Worldwide, only few studies related to Artiodactyla included testing for *Hepatozoon*, finding *H.* *canis* in camels from Saudi-Arabia [54] and in ticks collected from goats in China [55] and Romania [56] and *Hepatozoon* sp. in ticks collected from cattle in Pakistan [57]. To our knowledge, no information is available about *Hepatozoon* in wild Lagomorpha, except for a Spanish study in which no *Hepatozoon* was detected in European hares (*Lepus* *europaeus*) [35].

In rodents, *Hepatozoon* has been reported in Europe in Finland, Estonia and western Russia [58], Lithuania [59], Poland [60–62], Hungary [63], the Czech Republic [64], Slovakia [65], Great Britain [66–69], Germany [70, 71], Austria [72], Turkey [73] and Spain [32]. In rodent-related studies in which *Hepatozoon* could be identified to species level, in bank voles (*Myodes* = *Clethrionomys* *glareolus*) *H.* *erhardovae* was often detected [58, 60, 62, 63, 65, 68, 71, 72] and, to a lesser extent, *H.* *sciuri* in red squirrels (*Sciuri* *vulgaris*) [64], *H.* *griseisciuri* in gray squirrels (*Sciuris* *carolinensis*) [69] and *H.* *lavieri* in common voles (*Microtus* *arvalis*) [61].

Data about *Hepatozoon* in European ticks are also scarcely reported. It has long been believed that *Rhipicephalus* *sanguineus* sensu lato is the only known vector of *H.* *canis* in Europe and of many other *Hepatozoon* species [48, 51, 74, 75]. Recent findings of *Hepatozoon* sp. in other tick species raise questions about their vectorial role. Those findings include *H.* *canis* in *Ixodes* *ricinus*, *I.* *canisuga*, *I.* *hexagonus* and *Dermacentor* *reticulatus* ticks feeding on foxes in Germany [76], in *Haemaphysalis* *concinna* ticks collected from a dog in Poland [77] and in the abovementioned ticks (*I.* *ricinus*) from goats (and also dogs, fox and cat) in Romania [56]. Also, *H.* *canis* positive questing *I.* *ricinus* ticks were found in Slovakia and the Czech Republic [65] and in a *R.* *turanicus* collected from an infected fox in Italy [78].

Here, we aim to gain more knowledge on species distribution and prevalence of *Hepatozoon* among wild mammals and ticks in Europe. For this, we investigated a wide range of ungulates, carnivores and small mammals and ticks collected from animals and vegetation from five European countries. Animals and ticks were tested for the presence of *Hepatozoon* spp. using PCR and sequencing methods.

## Methods

### Mammals

For this study, animal samples originating from Belgium, the Netherlands, Austria, Bosnia and Herzegovina, and Croatia were used. From each animal a piece of spleen was collected for the survey. From gray wolves (*Canis* *lupus*) one or multiple samples were collected after a complete necropsy (see Additional file 3: Table S4 for detailed information). Animals were culled during regular or sanitary shooting in a period from 2010 to 2019, and no animal was shot for the purpose of this study only. All investigated gray wolves from the Netherlands were roadkill animals. All samples were collected within the frameworks of national game management and population control programs according to national laws. Samples from other mammals besides the gray wolves from the Netherlands and Belgium were gathered for previous studies [79] and used for *Hepatozoon* detection in this study.

Free-living rodent adults in Croatia were captured in Sherman live traps as described in an earlier study [80]. We followed animal experimentation guidelines approved by the American Society of Mammalogists [81]. Captured live animals were anesthetised in bags containing ether-soaked cotton. Deeply anesthetized animals were killed by cervical dislocation as described in the guidelines. Dead animals were aseptically dissected, and the tissue samples for DNA extraction were frozen at − 80 °C for several days before further analysis.

### Ticks

Questing ticks were collected by dragging vegetation and other environments, and ticks were collected from various animals originating from Croatia and the Netherlands. Ticks were washed and stored in 98% ethanol until further processing, after morphological identification to species level using morphological keys as described in [82, 83].

### DNA extraction, amplification and sequencing

DNA from animals was extracted from 10 mg of spleen and/or other organs (gray wolves) using DNA blood and tissue kit (Qiagen, Hilden, Germany) according to the manufacturer’s instructions.

For extraction of individual ticks’ DNA NucleoSpin® DNA Insect (Macherey Nagel) was used. The forward primer HepF 5’-ATACATGAGCAAAATCTCAAC-3’ and the reverse primer HepR 5’-CTTATTATTCCATGCTGCAG-3’ were used to amplify a fragment of ~ 670 bp of the 18S rRNA gene [84].

PCR reaction mixtures of 20 µl were prepared containing 10 µl G2 GOTaq mastermix (Promega, Madison, WI, USA), 7.2 µl DNase/RNase-Free distilled water (Promega), 0.4 µl 10 pmol/µl of each primer and 2 µl of sample. Positive (DNA of *H.* *canis* confirmed with sequencing from earlier studies) and negative (water from GoTaq G2 Mastermix) controls including extraction controls were used in all amplifications. The amplification product was analyzed using capillary electrophoresis on the QIAexcel system (QIAGEN, Hilden, Germany). For the purpose of further DNA sequencing, amplified PCR product was purified using ExoSAP-IT-PCR Clean-Up Reagent, according to the manufacturer’s instructions (USB Corporation, Cleveland, OH, USA). Sequencing in both directions was performed by Macrogen Europe with the same primers used for PCRs. The sequences were assembled using the SeqMan Pro software edited with EditSeq of the Lasergene software (DNASTAR, Madison WI, USA) and compared with available sequences using BLAST (https://blast.ncbi.nlm.nih.gov/Blast.cg) system analysis.

### Phylogenetic analysis

The 18S rRNA sequences of *Hepatozoon* obtained in this research and the deposited sequences of other *Hepatozoon* species and isolates available in the GenBank® were analyzed for phylogenetic relationships. The phylogenetic tree was subjected to an unweighted pair group method with arithmetic mean (UPGMA) clustering analysis using the tree builder tool incorporated in Geneious Prime (HKY). *Hepatozoon* sequences generated in this study were deposited in the NCBI GenBank® database under the accession numbers MH656727-MH656732 and KT274177-KT274186.

## Results

### Descriptive results

For this study, 2801 mammals and 754 ticks were tested for the presence of *Hepatozoon* with PCR. The mammals were order Artiodactyla (*n* = 1233), further divided in the Families Bovidae (*n* = 181), Suidae (*n* = 289) and Cervidae (*n* = 763); order Carnivora (*n* = 865), further divided in the Families Canidae (*n* = 336), Ursidae (*n* = 79), Mustelidae (*n* = 446) and Procyonidae (*n* = 4); order Eulipotyphia (*n* = 1), of which only the family Erinaceidae (*n* = 1); order Lagomorpha (*n* = 171), of which only the family Leporidae (*n* = 171) and order Rodentia (*n* = 531), further divided into the families Sciuridae (*n* = 53), Cricetidae (*n* = 167) and Muridae (*n* = 311). Of these 2801 animals, 36 originated from Bosnia and Herzegovina, 1186 from Croatia, 208 from Austria and 1371 from Belgium/the Netherlands. From Austria and Bosnia and Herzegovina, no Eulipotyphia, Lagomorpha or Rodentia were tested, and from the Netherlands/Belgium, no Eulipotyphia and Lagomorpha. Precise numbers of each mammal species and origin are given in Table 1 (Artiodactyla), Table 2 (Carnivora), Table 3 (small mammals; Rodentia, Eulipotyphia and Lagomorpha) and Additional file 1: Table S1 (all mammals).

**Table 1:** Presence of *Hepatozoon* spp. detected in samples of Artiodactyla

Animal species	Country	Animals (n)	*Hepatozoon* (n)	*Hepatozoon* (%)	*H.* *canis* MH656730	*H.* *martis* MH656728
Chamois *Rupicapra* *rupicapra*	Croatia	55	0	0		
	Austria	40	1	2.5		1
Alpine ibex *Capra* *ibex*	Austria	3	0	0		
Mouflon *Ovis* *orientalis* *musimon*	Croatia	62	0	0		
Mouflon *Ovis* *gmelini* *musimon*	Austria	21	0	0		
Roe deer *Capreolus* *capreolus*	Croatia	48	0	0		
	Austria	20	5	2.5	3	2
	Neth/Bel	462	0	0		
Red deer *Cervus* *elaphus*	Croatia	107	0	0		
	Austria	113	0	0		
Fallow deer *Dama* *dama*	Croatia	13	0	0		
Wild boar *Sus* *scrofa*	Croatia	254	0	0		
	Bosnia	35	0	0		
Total		1233	6	0.5	3	3

Species delineation was performed by comparison to sequences from GenBank®

*Bel* Belgium, *Neth* the Netherlands

**Table 2:** Presence of *Hepatozoon* spp. detected in samples from Carnivora

Animal species	Country	Animals (n)	*Hepatozoon* (n)	*Hepatozoon* (%)	*H.* *canis* MH656729	*H.* *canis* MH656730	*H.* *martis* MH656728
Golden jackal *Canis* *aureus*	Croatia	26	21	80.8	17	4	
Gray wolf *Canis* *lupus*	Croatia	120	65	54.2	43	22	
	Bosnia	1	1	100		1	
	Neth	8	8^*^	100	3	2	
Red fox *Vulpes* *vulpes*	Neth/Bel	174	76	43.7		76	
Raccoon dog *Nyctereutes* *procyonoides*	Austria	7	0	0			
Brown bear *Ursus* *arctos*	Croatia	79	0	0			
Badger *Meles* *meles*	Croatia	64	6	9.4	4	1	1
	Neth/Bel	99	0	0			
Stone marten *Martes* *foina*	Croatia	66	42	63.6			42
	Neth/Bel	67	32	47.8			32
Pine marten *Martes* *martes*	Neth	50	37	74.0			37
Europen polecat *Martes* *putorius*	Neth/Bel	100	10	10.0			10
Raccoon *Procyon* *lotor*	Austria	4	0	0			
Total		865	298	34.5	67	106	122

Species delineation was performed by comparison to sequences from GenBank®

^*^No sequences could be obtained from three samples. *Bel* Belgium, *Neth* the Netherlands

**Table 3:** Presence of *Hepatozoon* spp. detected in samples from small mammals (Rodentia, Eulipotyphia and Lagomorpha)

Animal species	Country	Animals (n)	*Hepatozoon* (n)	*Hepatozoon* (%)	*H.* *sciuri* MH656732	*Hepatozoon* sp. KT274179-86, MH656731	*H.* *ayorgbor* KT274177/8
Red squirrel *Sciuris* *vulgaris*	Neth/Bel	53	13	24.5	13		
Bank vole *Myodes* *glareolus*	Croatia	33	27	81.8		27	
	Neth/Bel	134	14	10.5		14	
Striped field mouse *Apodemus* *agrarius*	Croatia	2	0	0			
Yellow-necked mouse *Apodemus* *flavicollis*	Croatia	37	3	8.1		1	2 KT274178
Wood mouse *Apodemus* *sylvaticus*	Croatia	48	7	14.6		5	2 KT274177
	Neth/Bel	224	0	0			
Total Rodentia		531	64	12.1	13	47	4
European hedgehog *Erinaceus* *europaeus*	Croatia	1	1	100	1		
European hare *Lepus* *europaeus*	Croatia	171	1	0.6	1		

Species delineation was performed by comparison to sequences from GenBank®

*Bel* Belgium, *Neth* the Netherlands

In total 754 ticks of 15 tick species (Table 4) were used for this study. Of these, 287 originated from the Netherlands (all collected from animals), and the rest, 467 ticks, originated from Croatia (collected from animals *n* = 376, collected from the environment *n* = 91). Table 5 specifies the results of 38 ticks that were collected from three foxes, which tested negative for the presence of *Hepatozoon* DNA in their spleen samples.

**Table 4:** Prevalence and species of *Hepatozoon* in ticks collected from animals and environment (questing ticks)

Tick species	Ticks collected from (n)	*Hepatozoon* positives and spp. (GenBank® acc. no.)	Tick stage (n)
*Hyalomma* *marginatum*	Cow (30), goat (1), dog (2), horse (2)	0	
*Dermacentor* *reticulatus*	Cow (1), rabbit (1), bear (2), wild boar (1), vegetation (4)	0	
	Dog (3)	1 (*H.* *canis* MH656729)	Female
	Fox (9)	3 (*H.* *canis* MH656729)	Males (2), female
*Dermacentor* *marginatus*	Wild boar (3), dog (1)	0	
*Haemaphysalis* *concina*	Fox (1), vegetation (1)	0	
*Haemaphysalis* *punctata*	Sheep (1), horse (1), vegetation (6)	0	
*Haemaphysalis* *inermis*	Vegetation (6)	0	
*Haemaphysalis* *parva*	Vegetation (7)	0	
*Ixodes* *hexagonus*	Hedgehog (the Netherlands, 241), dog (4), cat (1)	0	
	Fox (13)	4 (*H.* *canis* MH656729)	Female (3), nymph (1)
		1	Female
*Ixodes* *ricinus*	Hedgehog (the Netherlands, 46), cat (1), roe deer (2), deer (2), vegetation (5)	0	
	Dog (20)	2	Female
	Horse (1)	1 (*H.* *canis* MH656729)	Female
	Fox (19)	6 (*H.* *canis* MH656729)	Male, female (5)
		3 (*H.* *canis* MH656730)	Females
	Vegetation (53)	1	Nymph
		1 (*H.* *martis* MH656728)	Male
*Ixodes* *canisuga*	Fox (29)	1 (*H.* *canis* MH656729)	Female
		2 (*H.* *canis* MH656729)	Female, nymph
		1	Female
	Vegetation (1)	0	
*Ixodes* *ventalloi*	Rabbit (6)	0	
	Fox (2)	1	Male
*Ixodes* *gibbosus*	Sheep (4)	0	
*Rhipicephalus* *turanicus*	Cow (14), sheep (66), goat (45), donkey (6), dog (7), vegetation (7)	0	
	Cat (3)	2 (*H.* *felis* MH656727)	Females
*Rhipicephalus* *bursa*	Cow (28), sheep (9), goat (19), donkey (1), vegetation (1)	0	
*Rhipicephalus* *sanguineus*	Sheep (1), goat (1)	0	
	Dog (13)	1 (*H.* *canis* MH656729)	Female
Total	754	31	

Ticks were collected in Croatia, unless stated otherwise

**Table 5:** *Hepatozoon* in ticks collected from negative foxes

Animal	Tick species	Number of ticks	Tick stage			*Hepatozoon* positives (GenBank® acc. no.)
			Larva	Nymph	Adult -female	
Fox 1	*Ixodes* *canisuga*	6			6	3 (*H.* *canis* MH656729)
	*Ixodes* *hexagonus*	5		2	3	0
Fox 2	*Ixodes* *canisuga*	13	0	12	1	9 nymph (*H.* *canis* MH656729)
	*Ixodes* *hexagonus*	5	1	2	2	1 female (*H.* *canis* MH656729)
						1 larva (*H.* *canis* MH656729)
Fox 3	*Ixodes* *canisuga*	5		2	3	1 female (*H.* *canis* MH656729)
						1 nymph (*H.* *canis* MH656729)
	*Ixodes* *hexagonus*	4		1	3	0

### Prevalence of *Hepatozoon* in mammals

See Tables 1, 2, 3 and Additional file 1: Table S1 for *Hepatozoon* prevalence results of 2801 tested mammals. Overall, 370 (13.2%) mammal samples tested positive for *Hepatozoon*. The Carnivora showed highest prevalence (34.8%), followed by Rodentia (12.1%). Artiodactyla and Lagomorpha showed the lowest *Hepatozoon* prevalence (0.5% and 0.6%, respectively). Within the Artiodactyla, only chamois (Bovidae) and roe deer (Cervidae) tested positive. The only animal that was tested within the order Eulipotyphia (an European hedgehog, *Erinaceus* *europeaus*) tested positive. Within the Carnivora, the four families showed differences in prevalence: the Canidae showed the highest prevalence (49.7%)*,* and within that family especially the golden jackals (80.8%), followed by gray wolves (over half of the tested wolves were positive) and red foxes (less than half of the tested foxes were positive). Within the Mustelidae, pine martens were most frequently infected, followed by stone martens and European polecats. In contrast, all samples of the Families Ursidae and Procyonidae of the Carnivora tested negative. Within the Rodentia, the Muridae showed much lower prevalence (3.2%) than Sciuridae (24.5%) and Cricetidae (24.6%).

Regarding origin, the mammals from Croatia (*n* = 1186) and the Netherlands/Belgium (*n* = 1371) showed higher prevalence (14.6% and 13.9%, respectively) than animals from Austria (*n* = 208) and Bosnia (*n* = 36) (2.9% and 2.8%, respectively). The chamois and roe deer (Artiodactyla) that tested positive, were all from Austria. It is interesting to point out the difference in prevalence of *Hepatozoon* among bank voles (Croatia 81.8% and the Netherlands/Belgium 10.5%) and wood mice (Croatia 14.6% and the Netherlands/Belgium 0.0%).

### Prevalence of *Hepatozoon* in ticks

Overall, 31 (4.1%) of the 754 collected ticks tested positive for *Hepatozoon* (Table 2). Ticks of the genera *Dermacentor* showed the highest prevalence (16.0%), followed by *Ixodes* (5.3%) and *Rhipicephalus* (1.4%). None of the ticks of the genera *Hyalomma* (*n* = 35) and *Haemaphysalis* (*n* = 23) and none of the ticks from the Netherlands (*n* = 287) tested positive. Ticks that were collected from animals (29/663, 4.4%) tested positive more often than ticks collected from the environment (2/91, 2.2%).

### Sequence results

**Fig. 1:**
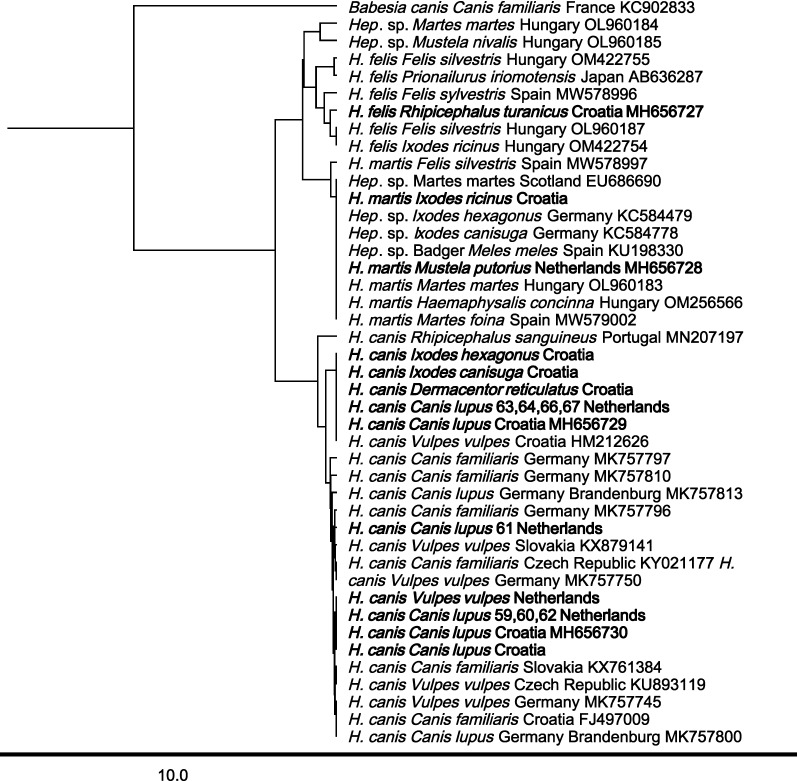
Phylogenetic tree (HKY, UPGMA with *Babesia* *canis* as outgroup) of *Hepatozoon* sequences from Carnivora. The sequences derived in this study are in bold

**Fig. 2:**
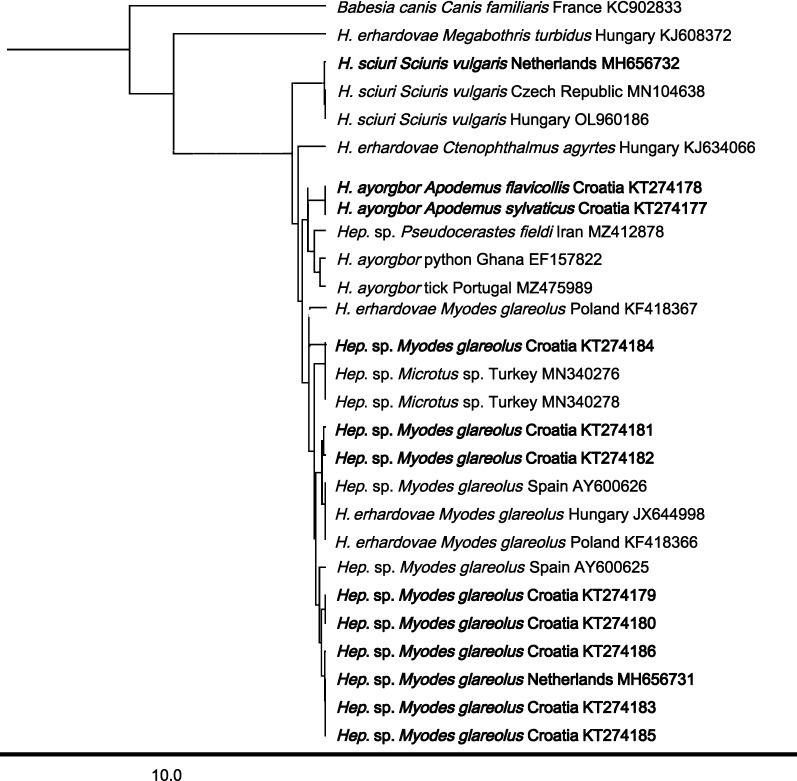
Phylogenetic tree (HKY, UPGMA with *Babesia* *canis* as outgroup) of *Hepatozoon* sequences from Rodentia. The sequences derived in this study are in bold

Results of the 18S sequence molecular analysis (Tables 1, 2, 3, 4, Figs. 1, 2, Additional file 1: Table S1, Additional file 2: Table S2) showed that five different *Hepatozoon* species were detected in the tested mammals and ticks. Also, *Hepatozoon* that could not be further specified to species level (*Hepatozoon* sp.) was found. Within all tested animals, *H.* *canis* was most prevalent (6.2%; 173/2801 mammals and 3.7%; 28/754 ticks). Interestingly, *H.* *canis* isolate MH656730 was most prevalent in mammals (3.8%; 107/2801) from Austria, Bosnia, Croatia and the Netherlands/Belgium, while *H.* *canis* isolate MH656729 was most often detected in ticks (2.5%; 19/754), and isolated solely from Croatian mammals (Tables 1, 2, 5 and Fig. 1). Furthermore, *H.* *canis* MH656729 was detected within the family Canidae (golden jackals and gray wolves), Mustelidae (badger), Erinaceaidae (European hedgehog) and Leporidae (European hare), while *H.* *canis* MH656730 was detected in carnivores, Canidae (golden jackals, gray wolves and red foxes), Mustelidae (badger), but also in Artiodactyla Cervidae (roe deer). Of the 38 ticks that were collected from *Hepatozoon*-negative foxes, in 16 (42.1%) ticks *H.* *canis* MH656729 was detected (Table 5). In Mustelidae, *H*. *martis* (MH656728) was most prevalent (96.1%; 122/127). In Sciuridae (squirrels), only *H*. *sciuri* (MH656732) was detected. In Rodentia, *Hepatozoon* sp. vole isolate (MH656731) was detected in bank voles (Cricetidae) from the Netherlands and Croatia and *H.* *ayorgbor* (EF157822) in yellow necked mice and wood mice (Fig. 2). Also, in bank voles, yellow-necked mice and wood mice from Croatia and the Netherlands, *Hepatozoon* sp. was detected (Table 1, Table 2, Fig. 2).

### Distribution of *Hepatozoon canis* in organs of gray wolves

Of the 119 (Croatia *n* = 114, the Netherlands *n* = 5) wolves of which different numbers of organs (range 1 to 9) were screened to assess the distribution of *H.* *canis*, 71 (Croatia *n* = 66, the Netherlands *n* = 5) had one or more positive organ(s) (Additional File 3; Table S4). *Hepatozoon* *canis* MH656729 was detected in organs of 50 wolves and *H.* *canis* MH656730 in organs of 19 wolves. Also, in two wolves, both *H.* *canis* isolates were detected. In most of the wolves, one (*n* = 18) or two (*n* = 34) organs were tested. Three and four organs were tested in six wolves each. Six organs were tested in four wolves, eight organs in one wolf and finally nine organs in two wolves. The organs that were most tested were spleen (*n* = 64), lymph node (*n* = 36) and skeletal muscle (*n* = 20). Bone marrow (*n* = 3) and blood (*n* = 1) were tested the least and brain (*n* = 7), myocardium (*n* = 10), lungs (*n* = 12), kidneys (*n* = 12) and liver (*n* = 13) in between.

Spleen samples tested positive most often (93.8%; 60/64), followed by samples of lungs (83.3%; 10/12), lymph nodes (75.0%; 27/36) and bone marrow (66.7%; 2/3). The seven brain samples and one blood sample tested negative. Also, myocardium (60.0%; 6/10), liver (53.9%; 7/13), kidney (41.7%; 5/12) and skeletal muscle (25.0%; 5/20) samples tested positive.

## Discussion

We investigated a wide range of mammal and tick species originating from five southeastern, central and western European countries for *Hepatozoon* prevalence and species diversity. *Hepatozoon* was detected in mammals from all five countries, with differences in prevalence. *Hepatozoon* prevalence of mammals in Austria and Bosnia and Herzogovina was ~ 3% compared to a prevalence in Croatia and Belgium/the Netherlands of 14–15%. The main reason for this difference in prevalence seems to be sampling bias. The animal species with the highest prevalence (*Canis* *aureus*, *C.* *lupus*, *Vulpes* *vulpes*, *Martes* *foina*, *M.* *martes* and *Myodes* *glareolus*; Tables 1, 2, 3, Additional file 1: Table S1) originated mainly from Croatia and Belgium/the Netherlands. From Austria and Bosnia and Herzegovina, fewer and different animal species were sampled (mainly Artilodactyla, a few Carnivora and no Rodentia). Since *Hepatozoon* is known to be present in Austrian [85] and Bosnian [40, 44, 86] carnivores and in Austrian rodents [72] and is most likely also present in Bosnian rodents, including samples of Carnivora and Rodentia from those countries, it probably would have increased prevalence.

High overall *Hepatozoon* prevalence was found in Carnivora, especially the Canidae and Mustelidae, and in Rodentia, especially the Cricetidae and Sciuridae, which is in accordance to other studies [41, 49, 63, 70, 87].

In this study, 670-bp fragments of the 18S rRNA gene were used for phylogenetic analysis and species determination. Even though for this purpose amplifications of longer fragments [88] or next-generation sequencing of nuclear, apicoplast and mitochondrial genes [89] are currently advised, we identified five different *Hepatozoon* species in the mammals and ticks: *H.* *canis* [90], *H.* *felis* [91] and the more recently named *H.* *martis* [44], *H.* *sciuri* [64] and *H.* *ayorgbor* [92]. Surprisingly, *H.* *martis* was not only detected in *Martes* *foina*, *M.* *martes* and other Mustelidae, but also in Artiodactyla (*R.* *rupicapra* and *C.* *capreolus*) from Austria. Also, in Austrian roe deer (*C.* *capreolus*), *H.* *canis* was detected. Broader host specificity is known for *H.* *martis* and *H.* *canis*, although the species were detected in other Carnivora, Canidae and Mustelidae [41]. The presence of *H.* *canis* in the spleen samples of roe deer and chamois therefore represents an unexpected finding. Although prevalence was low, current detection could suggest lack of host specificity as seen in other tick-borne apicomplexans, e.g. *Theileria* *capreoli* infecting gray wolves [93].

In the 31 positive (4.1%) ticks, only *Hepatozoon* species that are associated with Carnivores were detected (mainly *H.* *canis* and, to a lesser extent, *H.* *martis* and *H.* *felis*). This is not surprising, since *Hepatozoon* transmission is known to take place by carnivores (intermediate hosts) ingesting infected ticks (definitive hosts) [2]. We found *H.* *canis* not only in *R.* *sanguineus*, but also in *D.* *reticulatus*, *I.* *hexagonus*, *I.* *ricinus*, *I.* *canisuga* and *I.* *ventalloi*, which adds to the observations that other tick species than *R.* *sanguineus* may also be definitive tick hosts in the life cycle of *H.* *canis* [76, 94]. More research is necessary to confirm vector competence and capacity of these tick species for *Hepatozoon* since merely detection of the parasites in a tick is insufficient to designate the tick species as a vector.

In the Rodentia that were tested in our study, two *Hepatozoon* species could be identified: *H.* *sciuri* (in squirrels) and *H.* *ayorgbor* (in *A.* *flavicollis* and *A.* *sylvaticus*). The *Hepatozoon* sp. detected in bank voles (*M.* *glareolus*) from Croatia and the Netherlands could not be identified to species level. All *Hepatozoon* spp. sequences of bank voles from this study were (nearly) identical to each other and to *Hepatozoon* sequences from voles in GenBank® (Additional file 2: Table S3). As far as we know, these *Hepatozoon* sp. sequences were not found in any other mammalian species. Hence, we refer to this group as *Hepatozoon* sp. Vole isolate.

The species that were detected in rodents were not detected in the tested ticks. This could mean that ticks are not involved in the life cycle of these *Hepatozoon* species or that other tick species (e.g. *I.* *triangucileps*, which feeds only on small mammals [95]) and/or tick stages are involved. *Hepatozoon* *ayorgbor* has been described in snakes, ectoparasites and rodents, even though our finding in Croatian rodents (*A.* *flavicollis* and *A.* *sylvaticus*) is the first reported in European mammals. In the life cycle of *H.* *ayorgbor*, snakes can be infected via predation of rodents, with rodents serving as paratenic or intermediate hosts and mosquitoes as definitive invertebrate hosts [9], but findings in ticks and mites [96] suggest that other arthropods may be serving as definitive hosts as well. Whether the Portuguese tick (species unknown) in which *H.* *ayorgbor* was detected and of which the sequence from GenBank® (MZ475989) was used in our sequence analysis was truly infected or merely contaminated via blood feeding on an infected host is therefore not clear. *Hepatozoon* *ayorgbor*-like sequence was detected in three spleen samples of great gerbils in northwestern China [97], sharing 98.2% similarity to *H.* *ayorgbor* in the blood and liver of a ball python (*Python* *regius*) fed with tissues of mice experimentally infected with *H.* *ayorgbor* [9, 92]. Together with our findings, this confirms that rodents play a role in the life cycle of *H.* *ayorgbor*- or *H.* *ayorgbor*-related genotypes.

Our findings of *Hepatozoon* in carnivores and rodents from the Netherlands and Belgium are the first reported, but our study is also the first performed regarding detection of *Hepatozoon* in those countries. The species that were identified in mammals from the Netherlands and Belgium were *H.* *canis* in gray wolves and red foxes, *H.* *martis* in stone martens, pine martens and European polecats, and *H.* *sciuri* in squirrels. Even though a high prevalence of *Hepatozoon* was found in wild carnivores from the Netherlands/Belgium, to our knowledge, in The Netherlands no autochthonous *Hepatozoon* spillover from wild to domestic carnivores has been reported so far. No *Hepatozoon* was detected in the investigated Dutch and Belgian ticks. This could be because transmission of *H.* *canis* in for example foxes takes place via ticks in fox burrows (such as *I.* *canisuga* [98]) or via vertical transmission [18], which makes spillover to dogs less likely. To detect *H.* *canis* presence in ticks from the Netherlands and Belgium, other ticks than *I.* *hexagonus* and *I.* *ricinus* collected from hedgehogs would be advisable (Table 4).

The results of the investigated gray wolves show that spleen samples are most likely to test positive in case of a positive animal, which is in accordance with other reports [13].

## Conclusion

Our results show that *Hepatozoon* is widely present in wild mammals and ticks originating from several countries in West, Central and Southeast Europe. Presence of *Hepatozoon* was confirmed in ticks other than the ‘usual suspect’ *R.* *sanguineus*. Besides confirming presence of *Hepatozoon* in wild mammals and ticks in countries in which *Hepatozoon* was previously detected, presence of this tick-borne parasite in the Netherlands/Belgium was demonstrated for the first time, even though circulation in ticks could not be confirmed. Since spillover from wildlife to domestic animals in countries where *Hepatozoon* is endemic occurs, veterinary clinicians in the Netherlands/Belgium should be aware of the presence of this tick-borne disease.

## Supplementary Information

**Additional file 1: Table S1.** Overall prevalence of *Hepatozoon* and species in tested animal species and among countries.

**Additional file 2: Table S2**. *Hepatozoon* *ayorgbor* isolates from Croatian small wild rodents compared to the python isolate EF157822. **Table S3**. *Hepatozoon* sp. isolates infecting Croatian small wild rodents compared to Spanish *Myodes* *glareolus* isolates AY600625 and AY600626.

**Additional file 3: Table S4**. Distribution of *H.* *canis* in organs of gray wolves.

## Data Availability

*Hepatozoon* sequences generated in this study were submitted to NCBI GenBank® under accession numbers MH656727-MH656732 and KT274177-KT274186. Data supporting the conclusions of this article are included within the article and its additional files. A limited amount of DNA from samples is available upon reasonable request.
