# Supplementary material for: Diversity of Hepatozoon species in wild mammals and ticks in Europe
Source: Parasit Vectors. 2023 Jan 24;16:27. doi: 10.1186/s13071-022-05626-8 (PMC9872412; doi:10.1186/s13071-022-05626-8)
Supplement: Supplementary file 1 — Additional file 1: Table S1. Overall prevalence of Hepatozoon and species in tested animal species and among countries. [file 13071_2022_5626_MOESM1_ESM.docx]

**Additional file 1. Table S1.**

Overall prevalence of *Hepatozoon* and species in tested mammals and among countries. The most common isolates and their GenBank^®^ accession number are indicated in last six columns.

| Order | Family | Animal species | Country | No. of animals | No. *Hep.* pos. | *%* pos. | *H. canis* MH656729 | *H. canis* MH656730 | *H. martis* MH656728 | *H. sciuri* MH656732 | *Hepatozoon* sp. KT274179-86, MH656731 | *H. ayorgbor* KT274177/ KT274178 |
| --- | --- | --- | --- | --- | --- | --- | --- | --- | --- | --- | --- | --- |
| Artiodactyla (0.5%, 6/1233) | Bovidae (0.6%, 1/181) | Chamois *Rupicapra rupicapra* (1.1%, 1/95) | Croatia | 55 | 0 | 0 |  |  |  |  |  |  |
|  |  |  | Austria | 40 | 1 | 2.5 |  |  | 1 |  |  |  |
|  |  | Alpine ibex *Capra ibex* | Austria | 3 | 0 | 0 |  |  |  |  |  |  |
|  |  | Muflon *Ovis orientalis musimon* | Croatia | 62 | 0 | 0 |  |  |  |  |  |  |
|  |  | Muflon *Ovis gmelini musimon* | Austria | 21 | 0 | 0 |  |  |  |  |  |  |
|  | Suidae (0.0%, 0/289) | Wild boar *Sus scrofa* | Croatia | 254 | 0 | 0 |  |  |  |  |  |  |
|  |  |  | Bosnia | 35 | 0 | 0 |  |  |  |  |  |  |
|  | Cervidae (0.7%, 5/763) | Roe deer *Capreolus capreolus* (0.9%, 5/530) | Croatia | 48 | 0 | 0 |  |  |  |  |  |  |
|  |  |  | Austria | 20 | 5 | 2.5 |  | 3 | 2 |  |  |  |
|  |  |  | Neth/Bel | 462 | 0 | 0 |  |  |  |  |  |  |
|  |  | Red deer *Cervus elaphus* | Croatia | 107 | 0 | 0 |  |  |  |  |  |  |
|  |  |  | Austria | 113 | 0 | 0 |  |  |  |  |  |  |
|  |  | Fallow deer *Dama dama* | Croatia | 13 | 0 | 0 |  |  |  |  |  |  |
| Carnivora (34.5%, 298/865) | Canidae (49.70%, 163/328) | Golden jackal *Canis aureus* (80.8%) | Croatia | 26 | 21 | 80.8 | 17 | 4 |  |  |  |  |
|  |  | Gray wolf *Canis lupus* (54.6%, 66/121) | Croatia | 120 | 65 | 54.2 | 43 | 22 |  |  |  |  |
|  |  |  | Bosnia | 1 | 1 | 100 |  | 1 |  |  |  |  |
|  |  |  | Neth | 8 | 8 | 100 | 3 | 2 |  |  |  |  |
|  |  | Red fox *Vulpes vulpes* (43.7%) | Neth/Bel | 174 | 76 | 43.7 |  | 76 |  |  |  |  |
|  |  | Raccoon dog *Nyctereutes procyonoides* | Austria | 7 | 0 | 0 |  |  |  |  |  |  |
|  | Ursidae (0.0%) | Brown bear *Ursus arctos* | Croatia | 79 | 0 | 0 |  |  |  |  |  |  |
|  | Mustelidae (28.5%, 127/446) | European badger *Meles meles* (3.7%, 6/163) | Croatia | 64 | 6 | 9.4 | 4 | 1 | 1 |  |  |  |
|  |  |  | Neth/Bel | 99 | 0 | 0 |  |  |  |  |  |  |
|  |  | Stone marten *Martes foina* (55.6%, 74/133) | Croatia | 66 | 42 | 63.6 |  |  | 42 |  |  |  |
|  |  |  | Neth/Bel | 67 | 32 | 47.8 |  |  | 32 |  |  |  |
|  |  | Pine marten *Martes martes* (74.0%) | Neth | 50 | 37 | 74.0 |  |  | 37 |  |  |  |
|  |  | European polecat *Mustela putorius* (10.0%) | Neth/Bel | 100 | 10 | 10.0 |  |  | 10 |  |  |  |
|  | Procyonidae | Raccoon *Procyon lotor* | Austria | 4 | 0 | 0 |  |  |  |  |  |  |
| Eulipotyphia (100%) | Erinaceaidae | European hedgehog *Erinaceus europaeus* | Croatia | 1 | 1 | 100.0 | 1 |  |  |  |  |  |
| Lagomorpha (0.6%, 1/171) | Leporidae | European hare *Lepus europaeus* | Croatia | 171 | 1 | 0.6 | 1 |  |  |  |  |  |
| Rodentia (12.1%, 64/531) | Sciuridae (24.5%, 13/53) | Red squirrel *Sciurus vulgaris* | Neth/Bel | 53 | 13 | 24.5 |  |  |  | 13 |  |  |
|  | Cricetidae (24.6%, 41/167) | Bank vole *Myodes glareolus* | Croatia | 33 | 27 | 81.8 |  |  |  |  | 27 |  |
|  |  |  | Neth/Bel | 134 | 14 | 10.5 |  |  |  |  | 14 |  |
|  | Muridae (3.2%, 10/311) | Striped field mouse *Apodemus agrarius* | Croatia | 2 | 0 | 0 |  |  |  |  |  |  |
|  |  | Yellow neck mouse *Apodemus flavicollis* (8.1%, 3/73) | Croatia | 37 | 3 | 8.1 |  |  |  |  | 1 | 2 KT274178 |
|  |  | Wood mouse *Apodemus sylvaticus* (2.6%, 7/272) | Croatia | 48 | 7 | 14.6 |  |  |  |  | 5 | 2 KT274177 |
|  |  |  | Neth/Bel | 224 | 0 | 0 |  |  |  |  |  |  |
| Total | | | | 2.801 | 370 | 13.2 | 69 | 109 | 125 | 13 | 47 | 4 |

*Abbreviations*: Bel, Belgium; Neth, the Netherlands
