# Supplementary material for: Diversity of Hepatozoon species in wild mammals and ticks in Europe
Source: Parasit Vectors. 2023 Jan 24;16:27. doi: 10.1186/s13071-022-05626-8 (PMC9872412; doi:10.1186/s13071-022-05626-8)
Supplement: Supplementary file 2 — Additional file 2: Table S2. Hepatozoon ayorgbor isolates from Croatian small wild rodents compared to the python isolate EF157822. Table S3. Hepatozoon sp. isolates infecting Croatian small wild rodents compared to Spanish Myodes glareolus isolates AY600625 and AY600626. [file 13071_2022_5626_MOESM2_ESM.docx]

**Additional file 2: Table S2 and S3**

**Table S2.** *H. ayorgbor* isolates from Croatian small wild rodents, compared to the python isolate (Acc. No. EF157822). *H. ayorgbor* sequences are distributed into two groups, according to the single point mutation found. Representative sequence for each group have been submitted to the GenBank^®^, and their Accession numbers are presented in the last column. Position numbering is according to the isolate EF157822 from python.

| **Isolate group** | **Isolate/rodent label** | **Nucleotide at the pos. 153** | **Acc. No.** |
| --- | --- | --- | --- |
|  | EF157822 | A | **EF157822** |
| **1** | 55, 73 | A | **KT274177** |
| **2** | 53, 74 | G | **KT274178** |

**Table S3.** *Hepatozoon sp.* isolates infecting Croatian small wild rodents. *Hepatozoon sp*. sequences are highly similar to the Spanish isolates from *Myodes glareolus* (Acc. Nos. AY600625 and AY600626). *Hepatozoon sp*. sequences are distributed into four groups, according to individual point mutations found. Representative sequences for each group have been submitted to the GenBank^®^, and their Accession numbers are presented in the last column. Position numbering is according to the isolates AY600625 and AY600626 from *Myodes glareolus*.

|  | | **Mutation positions** | | | | | | | | |  |
| --- | --- | --- | --- | --- | --- | --- | --- | --- | --- | --- | --- |
| **Isolate group** | **Isolates/rodent labels** | **172** | **174** | **221** | **591** | **624** | **698** | **712** | **742** | **745** | **Acc. No.** |
|  | BV2 | C | T | A | A | T | C | A | A | A | **AY600625** |
|  | BV1 | C | T | G | A | T | T | G | G | T | **AY600626** |
| **1** | 40, 44, 58, 60, 61, 64 | A | A | G | A | T | T | G | G | T | **KT274181**  **KT274182** |
| **2** | 49, 67 | A | A | G | A | T | C | G | A | A | **KT274179**  **KT274180** |
| **3** | 37, 50 | A | A | A | T | C | C | A | A | A | **KT274183**  **KT274184** |
| **4** | 38, 39, 41, 42, 45-48, 51, 52, 54, 56, 57, 59, 62, 63, 65, 66, 68-72 | A | A | A | A | T | C | A | A | A | **KT274185**  **KT274186** |
