# Supplementary material for: Diversity of Hepatozoon species in wild mammals and ticks in Europe
Source: Parasit Vectors. 2023 Jan 24;16:27. doi: 10.1186/s13071-022-05626-8 (PMC9872412; doi:10.1186/s13071-022-05626-8)
Supplement: Supplementary file 3 — Additional file 3: Table S4. Distribution of H. canis in organs of gray wolves. [file 13071_2022_5626_MOESM3_ESM.docx]

**Additional file 3. Table S4.** Distribution of *H. canis* in organs of gray wolves. All animals originate from Croatia, except the ones in which NL (the Netherlands) is in the Animal ID.

| **Animal ID** | **Genotype *H. canis*** | **Lungs** | **Kidney** | **Spleen** | **Blood** | **Lymph node** | **Myo-cardium** | **Skeletal muscle** | **Brain** | **Liver** | **Bone marrow** | **No. of positive organs** | **No. of included organs** | **Positive organ** |
| --- | --- | --- | --- | --- | --- | --- | --- | --- | --- | --- | --- | --- | --- | --- |
| WCRO060 | MH656729 | n.d. | n.d. | 1 | n.d. | n.d. | n.d. | n.d. | n.d. | n.d. | n.d. | 1 | 1 | spleen |
| WCRO114 | MH656729 | n.d. | n.d. | 1 | n.d. | n.d. | n.d. | n.d. | n.d. | n.d. | n.d. | 1 | 1 | spleen |
| WCRO124 | MH656729 | n.d. | n.d. | 1 | n.d. | n.d. | n.d. | n.d. | n.d. | n.d. | n.d. | 1 | 1 | spleen |
| WCRO130 | MH656730 | n.d. | n.d. | n.d. | n.d. | 1 | n.d. | n.d. | n.d. | n.d. | n.d. | 1 | 1 | lymph node |
| WCRO131 | MH656729 | n.d. | n.d. | 1 | n.d. | n.d. | n.d. | n.d. | n.d. | n.d. | n.d. | 1 | 1 | spleen |
| WCRO133 | MH656729 | n.d. | n.d. | 1 | n.d. | n.d. | n.d. | n.d. | n.d. | n.d. | n.d. | 1 | 1 | spleen |
| WCRO134 | MH656729 | n.d. | n.d. | 1 | n.d. | n.d. | n.d. | n.d. | n.d. | n.d. | n.d. | 1 | 1 | spleen |
| WCRO142 | MH656730 | n.d. | n.d. | 1 | n.d. | n.d. | n.d. | n.d. | n.d. | n.d. | n.d. | 1 | 1 | spleen |
| WCRO151 | MH656729 | n.d. | n.d. | n.d. | n.d. | 1 | n.d. | n.d. | n.d. | n.d. | n.d. | 1 | 1 | lymph node |
| WCRO164 | MH656730 | n.d. | n.d. | n.d. | n.d. | 1 | n.d. | n.d. | n.d. | n.d. | n.d. | 1 | 1 | lymph node |
| V33 | MH656729 | n.d. | n.d. | 1 | n.d. | n.d. | n.d. | n.d. | n.d. | n.d. | n.d. | 1 | 1 | spleen |
| V34 | MH656730 | n.d. | n.d. | 1 | n.d. | n.d. | n.d. | n.d. | n.d. | n.d. | n.d. | 1 | 1 | spleen |
| V36 | MH656729 | n.d. | n.d. | 1 | n.d. | n.d. | n.d. | n.d. | n.d. | n.d. | n.d. | 1 | 1 | spleen |
| V37 | MH656729 | n.d. | n.d. | 1 | n.d. | n.d. | n.d. | n.d. | n.d. | n.d. | n.d. | 1 | 1 | spleen |
| V41 | MH656729 | n.d. | n.d. | 1 | n.d. | n.d. | n.d. | n.d. | n.d. | n.d. | n.d. | 1 | 1 | spleen |
| V42 | MH656729 | n.d. | n.d. | 1 | n.d. | n.d. | n.d. | n.d. | n.d. | n.d. | n.d. | 1 | 1 | spleen |
| V47 | MH656729 | n.d. | n.d. | 1 | n.d. | n.d. | n.d. | n.d. | n.d. | n.d. | n.d. | 1 | 1 | spleen |
| WCRO033 | MH656729 | n.d. | 1 | n.d. | n.d. | n.d. | n.d. | n.d. | n.d. | n.d. | n.d. | 1 | 1 | kidney |
| WCRO032 | MH656729 | n.d. | n.d. | 1 | n.d. | n.d. | n.d. | 0 | n.d. | n.d. | n.d. | 1 | 2 | spleen |
| WCRO086 | MH656729 | n.d. | n.d. | 1 | n.d. | n.d. | n.d. | 0 | n.d. | n.d. | n.d. | 1 | 2 | spleen |
| WCRO087 | MH656730 | n.d. | n.d. | 1 | n.d. | n.d. | n.d. | 0 | n.d. | n.d. | n.d. | 1 | 2 | spleen |
| WCRO129 | MH656729 | n.d. | n.d. | 1 | n.d. | n.d. | n.d. | 0 | n.d. | n.d. | n.d. | 1 | 2 | spleen |
| WCRO136 | MH656730 | n.d. | n.d. | 1 | n.d. | 0 | n.d. | n.d. | n.d. | n.d. | n.d. | 1 | 2 | spleen |
| WCRO140 | MH656729 | n.d. | n.d. | 1 | n.d. | 0 | n.d. | n.d. | n.d. | n.d. | n.d. | 1 | 2 | spleen |
| WCRO146 | MH656729 | n.d. | n.d. | 1 | n.d. | 0 | n.d. | n.d. | n.d. | n.d. | n.d. | 1 | 2 | spleen |
| WCRO152 | MH656730 | n.d. | n.d. | 1 | n.d. | 0 | n.d. | n.d. | n.d. | n.d. | n.d. | 1 | 2 | spleen |
| WCRO153 | MH656729 | n.d. | n.d. | 1 | n.d. | 0 | n.d. | n.d. | n.d. | n.d. | n.d. | 1 | 2 | spleen |
| WCRO154 | MH656730 | n.d. | n.d. | 0 | n.d. | 1 | n.d. | n.d. | n.d. | n.d. | n.d. | 1 | 2 | lymph node |
| WCRO155 | MH656729 | n.d. | n.d. | 1 | n.d. | 0 | n.d. | n.d. | n.d. | n.d. | n.d. | 1 | 2 | spleen |
| WCRO158 | MH656729 | n.d. | n.d. | 1 | n.d. | 0 | n.d. | n.d. | n.d. | n.d. | n.d. | 1 | 2 | spleen |
| WCRO170 | MH656729 | n.d. | n.d. | 1 | n.d. | 0 | n.d. | n.d. | n.d. | n.d. | n.d. | 1 | 2 | spleen |
| WCRO167 | MH656729 | n.d. | n.d. | 0 | n.d. | 1 | n.d. | n.d. | n.d. | n.d. | n.d. | 1 | 2 | lymph node |
| WCRO126 | MH656730 | n.d. | n.d. | 1 | n.d. | 1 | n.d. | n.d. | n.d. | n.d. | n.d. | 2 | 2 | spleen, lymph node |
| WCRO132 | MH656730 | n.d. | n.d. | 1 | n.d. | 1 | n.d. | n.d. | n.d. | n.d. | n.d. | 2 | 2 | spleen, lymph node |
| WCRO135 | MH656729 | n.d. | n.d. | 1 | n.d. | 1 | n.d. | n.d. | n.d. | n.d. | n.d. | 2 | 2 | spleen, lymph node |
| WCRO137 | MH656729 | n.d. | n.d. | 1 | n.d. | 1 | n.d. | n.d. | n.d. | n.d. | n.d. | 2 | 2 | spleen, lymph node |
| WCRO139 | MH656729 | n.d. | n.d. | 1 | n.d. | 1 | n.d. | n.d. | n.d. | n.d. | n.d. | 2 | 2 | spleen, lymph node |
| WCRO150 | MH656729 | n.d. | n.d. | 1 | n.d. | 1 | n.d. | n.d. | n.d. | n.d. | n.d. | 2 | 2 | spleen, lymph node |
| WCRO156 | MH656729 | n.d. | n.d. | 1 | n.d. | 1 | n.d. | n.d. | n.d. | n.d. | n.d. | 2 | 2 | spleen, lymph node |
| WCRO159 | MH656729 | n.d. | n.d. | 1 | n.d. | 1 | n.d. | n.d. | n.d. | n.d. | n.d. | 2 | 2 | spleen, lymph node |
| WCRO160 | MH656730 | n.d. | n.d. | 1 | n.d. | 1 | n.d. | n.d. | n.d. | n.d. | n.d. | 2 | 2 | spleen, lymph node |
| WCRO161 | MH656730 | n.d. | n.d. | 1 | n.d. | 1 | n.d. | n.d. | n.d. | n.d. | n.d. | 2 | 2 | spleen, lymph node |
| WCRO163 | MH656730 | n.d. | n.d. | 1 | n.d. | 1 | n.d. | n.d. | n.d. | n.d. | n.d. | 2 | 2 | spleen, lymph node |
| WCRO165 | MH656730 | n.d. | n.d. | 1 | n.d. | 1 | n.d. | n.d. | n.d. | n.d. | n.d. | 2 | 2 | spleen, lymph node |
| WCRO166 | MH656729 | n.d. | n.d. | 1 | n.d. | 1 | n.d. | n.d. | n.d. | n.d. | n.d. | 2 | 2 | spleen, lymph node |
| WCRO168 | MH656729 | n.d. | n.d. | 1 | n.d. | 1 | n.d. | n.d. | n.d. | n.d. | n.d. | 2 | 2 | spleen, lymph node |
| WCRO172 | MH656729 | n.d. | n.d. | 1 | n.d. | 1 | n.d. | n.d. | n.d. | n.d. | n.d. | 2 | 2 | spleen, lymph node |
| NL3170303030 | MH656730 | n.d. | n.d. | 1 | n.d. | n.d. | 1 | n.d. | n.d. | n.d. | n.d. | 2 | 2 | spleen (60), myocardium (59)* |
| NL3171113025 | MH656730 | n.d. | n.d. | 1 | n.d. | n.d. | 1 | n.d. | n.d. | n.d. | n.d. | 2 | 2 | spleen (62), myocardium (61)* |
| NL3200304040 | MH656729 | n.d. | n.d. | 1 | n.d. | n.d. | 1 | n.d. | n.d. | n.d. | n.d. | 2 | 2 | spleen (64), myocardium (63)* |
| NL3210307001 | MH656729 | n.d. | n.d. | 1 | n.d. | n.d. | 1 | n.d. | n.d. | n.d. | n.d. | 2 | 2 | spleen, myocardium (66)* |
| NL3210529001 | MH656729 | n.d. | n.d. | 1 | n.d. | n.d. | 1 | n.d. | n.d. | n.d. | n.d. | 1 | 2 | spleen (67)* |
| WCRO040 | MH656730 | n.d. | n.d. | 1 | n.d. | n.d. | n.d. | n.d. | 0 | 0 | n.d. | 1 | 3 | spleen |
| WCRO096 | MH656729 | 1 | 0 | n.d. | n.d. | n.d. | n.d. | 0 | n.d. | n.d. | n.d. | 1 | 3 | lungs |
| WCRO112 | MH656729 | n.d. | 0 | n.d. | n.d. | n.d. | n.d. | 0 | n.d. | 1 | n.d. | 1 | 3 | liver |
| WCRO106 | MH656729 | 1 | 1 | n.d. | n.d. | n.d. | n.d. | 0 | n.d. | n.d. | n.d. | 2 | 3 | lungs, kidney |
| WCRO141 | MH656729 | n.d. | n.d. | 1 | n.d. | 1 | n.d. | 0 | n.d. | n.d. | n.d. | 2 | 3 | spleen, lymph node |
| WCRO084 | MH656730 | n.d. | n.d. | 1 | n.d. | 1 | n.d. | n.d. | n.d. | 1 | n.d. | 3 | 3 | spleen, lymph node, liver |
| WCRO024 | MH656729 | n.d. | n.d. | 1 | n.d. | n.d. | n.d. | n.d. | 0 | 0 | 0 | 1 | 4 | spleen |
| WCRO081 | MH656730 | n.d. | 0 | 1 | n.d. | n.d. | n.d. | 0 | 0 | n.d. | n.d. | 1 | 4 | spleen |
| WCRO147 | MH656729 | n.d. | n.d. | 1 | n.d. | 0 | n.d. | 0 | 0 | n.d. | n.d. | 1 | 4 | spleen |
| WCRO100 | MH656729 | 1 | n.d. | 1 | n.d. | n.d. | n.d. | 0 | n.d. | 0 | n.d. | 2 | 4 | lungs, spleen |
| WCRO071 | MH656729 | 1 | n.d. | 1 | n.d. | n.d. | n.d. | 0 | n.d. | 1 | n.d. | 3 | 4 | lungs, spleen, liver |
| WCRO119 | MH656729 | 1 | 1 | 1 | n.d. | n.d. | n.d. | 1 | n.d. | n.d. | n.d. | 4 | 4 | lungs, kidney, spleen,muscle |
| WCRO92 | MH656730 | 1 | 0 | 0 | n.d. | n.d. | 0 | 0 | n.d. | 1 | n.d. | 2 | 6 | lungs, liver |
| WCRO090 | MH656729 | 1 | n.d. | 1 | n.d. | 1 | 1 | 1 | n.d. | 0 | n.d. | 5 | 6 | lungs, spleen, lymph node, myocardium, muscle |
| WCRO127 | MH656729 | 1 | 1 | 1 | n.d. | 1 | n.d. | 1 | n.d. | 1 | n.d. | 6 | 6 | lungs, kidney, spleen,lymph node, muscle, liver |
| WCRO248 | MH656729 | 1 | 1 | 1 | n.d. | n.d. | n.d. | 1 | n.d. | 1 | 1 | 6 | 6 | lungs, kidney, spleen, muscle, liver, bone marrow |
| WCRO258 | MH656729 (sp.) MH656730 (l.n.) | 0 | 0 | 1 | n.d. | 1 | 0 | 0 | 0 | 0 | n.d. | 2 | 8 | spleen, lymph node |
| WCRO257 | MH656729 | 0 | 0 | 0 | 0 | 1 | 0 | 0 | 0 | 0 | n.d. | 1 | 9 | lymph node |
| WCRO255 | MH656729 (liv., b.m.) | 1 | 0 | 1 | n.d. | 1 | 0 | 1 | 0 | 1 | 1 | 6 | 9 | lungs, spleen, lymph node, muscle, liver, bone marrow |

*Abbreviations*: n.d., not done; 1, detected; 0, not detected; sp., spleen; l.n., lymph node; liv., liver; b.m., bone marrow; sk. musc., skeletal muscle

* the numbers in brackets refer to *Canis lupus* from the Netherlands in Figure 1.
